# Plasminogen Activator Inhibitor-1 Regulates LPS Induced Inflammation in Rat Macrophages through Autophagy Activation

**DOI:** 10.1155/2014/189168

**Published:** 2014-07-13

**Authors:** Zhong-Hui Wang, Wei-Ying Ren, Lei Zhu, Li-Juan Hu

**Affiliations:** ^1^Department of Pulmonary Medicine, Zhongshan Hospital, Fudan University, No. 180, Feng Lin Road, Shanghai 200032, China; ^2^Department of Geriatrics, Zhongshan Hospital, Fudan University, No. 180, Feng Lin Road, Shanghai 200032, China

## Abstract

*Background*. The mechanisms by which plasminogen activator inhibitor-1 (PAI-1) regulates inflammation, especially in acute respiratory distress syndrome (ARDS), are largely unknown. *Objective*. To assess the relationship between PAI-1 and autophagy in inflammatory reactions induced by LPS in rat NR8383 cells. *Methods*. ELISA was used to assess the amounts of TNF-*α*, IL-1*β*, and PAI-1 in cell culture supernatants; TLR4, MyD88, PAI-1, LC3, Beclin1, and mTOR protein and mRNA levels were determined by western blot and quantitative RT-PCR, respectively; western blot was used to determine NF-*κ*B protein levels. To further evaluate the role of PAI-1, the PAI-1 gene was downregulated and overexpressed using the siRNA transfection technology and the pCDH-PAI-1, respectively. Finally, the GFP Positive Expression Rate Method was used to determine the rate of GFP-LC3 positive NR8383 cells. *Results*. In LPS-induced NR8383 cells, TNF-*α*, IL-1*β*, and PAI-1 expression levels increased remarkably. Upon PAI-1 knockdown, TNF-*α*, IL-1*β*, PAI-1, TLR4, MyD88, NF-*κ*B, LC3, and Beclin1 levels were decreased, while mTOR increased. Conversely, overexpression of PAI-1 resulted in increased amounts of TNF-*α*, IL-1*β*, PAI-1, TLR4, MyD88, NF-*κ*B, LC3, and Beclin1. However, no significant change was observed in mTOR expression. *Conclusions.* In NR8383 cells, PAI-1 contributes in the regulation of LPS-induced inflammation, likely by promoting autophagy.

## 1. Introduction

Acute respiratory distress syndrome (ARDS) is a common life-threatening cause of acute respiratory failure that arises from a variety of local and systemic insults [1]. Despite some improvement in mortality with a lung-protective ventilator strategy [2], both morbidity and mortality remain high [3].

Plasminogen activator inhibitor-1 (PAI-1) is a member of the serine protease inhibitor (serpin) family. PAI-1 participates in decreasing plasmin generation and fibrinolytic potential by covalently binding to tissue-type plasminogen activator (tPA) and urokinase-type plasminogen activator (uPA) [4]. Recently, the effect of PAI-1 on inflammation has been described in addition to its classic role in fibrinolysis inhibition; it was shown that PAI-1 regulates the expression of some cytokines, including tumor necrosis factor (TNF)-*α*, interleukin (IL)-6, and interferon (IFN)-*γ* in lungs [5]. Higher PAI-1 levels are associated with increased mortality and reduced ventilator-free days among pediatric patients with acute lung injury (ALI), a less severe form of ARDS [6]. These findings suggest a role for impaired fibrinolysis in ALI pathogenesis in pediatric patients and that PAI-1 may serve as a useful biomarker for prognosis in patients with ALI [6]. Beside its role in fibrinolysis and coagulation, PAI-1 also acts as acute-phase proteins (APPs) that are rapidly upregulated following infectious and/or noninfectious injuries [7].

There are multiple cellular sources of plasminogen activator (PA) and PAI-1 that may be relevant to human ARDS. Unstimulated alveolar macrophages are profibrinolytic; primary isolates of human alveolar macrophages have PA activity and degrade a fibrin matrix in the presence of plasminogen [8]. By contrast, endotoxin stimulated alveolar macrophages inhibit fibrinolysis through an increase in PAI-1 activity [9, 10]. These findings suggest a decrease in the fibrinolytic activity of alveolar macrophages (reduced PA and increased PAI-1 activities) upon exposure of the lung to injurious stimuli. Excessive fibrin deposition enhances inflammatory responses by activating endothelial cells to produce proinflammatory mediators and increasing lung vascular permeability [11]. Therefore, assessing the extent of fibrinolysis in ALI may be important from a pathogenetic and prognostic standpoint [12]. The mechanisms by which PAI-1 contributes to inflammation have not been fully delineated. Interestingly, it was suggested that autophagy may contribute to the development of ARDS in H5N1 influenza patients [13].

Autophagy is a conserved and tightly regulated cellular catabolic process that involves the lysosomal degradation pathway [14, 15]. By selectively recycling macromolecules and organelles, autophagy is an integral part of normal cellular function that helps cells survive under starvation conditions to maintain cell growth and the development and homeostasis of organisms [16].

The relationships between autophagy, inflammation, and the activation of toll-like receptor (TLR) signaling pathways have been recently described [17]. In macrophages, LPS (lipopolysaccharide, often released from Gram-negative bacteria) was proposed to induce autophagy through TLR4, involving the adaptor, TRIF, receptor-interacting protein 1, and the p38 mitogen-activated protein kinase signaling pathway [18]. The activation of autophagy in macrophages in response to TLR4 activation depends on ROS production from the activation of the phagocytic reduced nicotinamide adenine dinucleotide phosphate oxidase 2 [19]. The processes are expected to take place in the lung as well, in particular in alveolar macrophages, which are important inflammatory cells in ARDS. Therefore, we aimed in this work to determine the relationship between PAI-1 and autophagy in inflammatory reactions induced by LPS in alveolar macrophages.

Using NR8383 rat macrophages induced by LPS, we found that TNF-*α*, IL-1*β*, TLR4, MyD88, and NF-*κ*B levels were decreased upon PAI-1 silencing. In addition, the levels of the autophagy markers LC3 and Beclin1 were reduced while mTOR (an autophagy inhibitor) was upregulated. Overexpression of PAI-1 produced opposite effects except for mTOR, where no significant change was observed. Overall, our findings suggested that PAI-1 regulates LPS-induced inflammation through activation of autophagy.

## 2. Materials and Methods

### 2.1. Cell Culture and Transfection

Rat alveolar NR8383 cells were purchased from the American Type Culture (ATCC, USA) and cultured at 37°C in a humidified environment containing 5% CO_2_ in F-12K medium (Gibco, USA), supplemented with 20% heat-inactivated fetal calf serum (Invitrogen, USA), 100 U/mL penicillin, and 100 *μ*g/mL streptomycin.

For gene silencing, logarithmic growth phase NR8383 cells were seeded in 6-well plates at a density of 5 × 10^5^ cells/well. Three siRNA pairs against rat PAI-1 mRNA (PAI1-siRNA1, PAI1-siRNA2, and PAI1-siRNA3) and a normal control siRNA (NC-siRNA) (Table 1) were synthesized by Shanghai Sangon Biotech (China) and transfected into NR8383 cells using the Lipofectamine 2000 transfection reagent (Invitrogen, USA), according to the manufacturer's instructions. After 24 hours of incubation in serum-free F-12K medium, the effectiveness of siRNAs in inhibiting PAI-1 expression was evaluated by real-time reverse transcriptase-polymerase chain reaction (RT-PCR).

On the other hand, the plasmid pCDH-PAI-1 encoding PAI-1 (Shanghai BioSune, China) was transfected into NR8383 cells using Lipofectamine 2000, and PAI-1 mRNA expression was upregulated after 24 h incubation, as assessed by RT-PCR.

### 2.2. Quantitative RT-PCR

Normal and transfected NR8383 cells were cultured in serum-free F-12K or serum-free F-12K containing 10 *μ*g/mL LPS for 4 hours. Total RNA was extracted from harvested cells using the TRIzol reagent (Sigma, USA), according to manufacturer's instructions. Reverse transcription (RT) was performed with a High Capacity cDNA Reverse Transcription Kit (TAKARA, JAPAN), following the manufacturer's directions. Quantitative real-time PCR was carried out using FastStart Universal SYBR Green Master (Bioteke Corporation, Beijing, China) on an ABI 7500 SDS RT-PCR system (Applied Biosystems, Foster City, CA, USA). The reaction conditions were 40 cycles of two-stage PCR consisting of denaturation at 95°C for 30 s and annealing at 60°C for 30 s after an initial denaturation step at 95°C for 15 s. Real-time PCR data were analyzed with the 2^−ΔΔCt^ method using GAPDH cDNA as endogenous control for normalization. The primers used for each gene are summarized in Table 2.

### 2.3. Preparation of Nuclear Proteins

NR8383 cells were treated as described above, washed three times with PBS, and resuspended in hypotonic buffer (10 mM Tris-HCl, pH 7.5 containing 10 mM KCl, 1.5 mM MgCl_2_, 0.1% Nonidet P-40, 1 mM DTT, 10 *μ*g/mL aprotinin, 10 *μ*g/mL leupeptin, 1 mM PMSF, and 1 mM Na_3_VO_4_). Cell lysates were incubated on ice with occasional mixing for 15 min followed by centrifugation at 1600 g for 10 min at 4°C, for separation of nuclei and cytosol. The nuclei (pellet) were resuspended in hypertonic buffer (20 mM Tris-HCl, 20% glycerol, 500 mM NaCl, 1.5 mM MgCl_2_, 0.2 mM EDTA, 1 mM DTT, 10 *μ*g/mL aprotinin, 10 *μ*g/mL leupeptin, 1 mM PMSF, and 1 mM Na_3_VO_4_) and incubated on ice with occasional mixing for 30 min. Finally, nuclear proteins were obtained by centrifugation at 15,000 g for 20 min at 4°C and used to evaluate NF-*κ*B amounts.

### 2.4. Western Blot Analysis

At 48 h after transfection with siRNA or pCDH-PAI-1, 10 *μ*g/mL LPS was added to cell culture media and incubated for 4 hours. Total protein samples were separated by 12% sodium dodecyl sulfate-polyacrylamide gel electrophoresis (SDS-PAGE) and transferred onto polyvinylidene difluoride (PVDF) filters (Millipore, USA). After blocking, membranes were probed with anti-rat monoclonal antibodies raised in mice against LC3, PAI-1, -TLR4, MyD88, Beclin1, mTOR, and NF-*κ*B (Abcam, USA) overnight at 4°C. Afterwards, membranes were incubated with horseradish peroxidase-conjugated anti-mouse antibody (Santa Cruze, USA) and detected by enhanced chemiluminescence (ECL) ChemiQ 3650mini provided by Bioshine (Shanghai, China). Equal protein loading was confirmed by reprobing blots with 0.5 *μ*g/mL GAPDH mouse anti-rat polyclonal antibodies (CST, USA). The integral optical density (IOD) of each band was measured using ImageJ software (National Institutes of Health, USA).

### 2.5. Enzyme-Linked Immunosorbent Assay (ELISA)

Normal and transfected NR8383 cells were cultured in serum-free F-12K or serum-free F-12K containing 10 *μ*g/mL LPS for 4 hours. Afterwards, supernatants were collected and TNF-*α*, IL-1*β*, and PAI-1 were quantified with specific ELISA kits supplied by R&D (Shanghai, China) as per the manufacturer's instructions.

### 2.6. Quantitation of GFP Positive Cells

Logarithmic growth phase NR8383 cells were seeded at a density of 1 × 10^5^ cells/well into 24-well plate containing cover glasses soaked in 75% sulfuric acid overnight and thoroughly washed and allowed 24 h for attachment. Then, 4 *μ*g GFP-LC3 plasmid (Shanghai Genechem, China) was transfected into cells with 10 *μ*L Lipofectamine 2000 (Invitrogen), according to manufacturer's instructions. Plasmids for PAI-1 silencing or overexpressing, and respective controls (described above) were cotransfected at the same time.

48 hours after transfection, cells were stimulated with 10 *μ*g/mL LPS for 4 hours. Thereafter, cells were placed in F-12K medium supplemented with 20% fetal calf serum and incubated for 24 hours. Micrographs were obtained on an Olympus confocal laser scanning microscope (Olympus, Japan) and the percentage of fluorescent cells was assessed.

### 2.7. Statistical Analysis

Data are presented as means ± SD of values obtained from at least three separate experiments by GraphPad 5.0. Comparisons were performed by one-way analysis of variance (ANOVA) with Tukey's test for multiple comparisons. Differences were considered significant at *P* < 0.05.

## 3. Results

### 3.1. Successful Regulation of the PAI-1 Gene by Transfection with siRNA and pCDH-PAI-1

**Figure 1:**
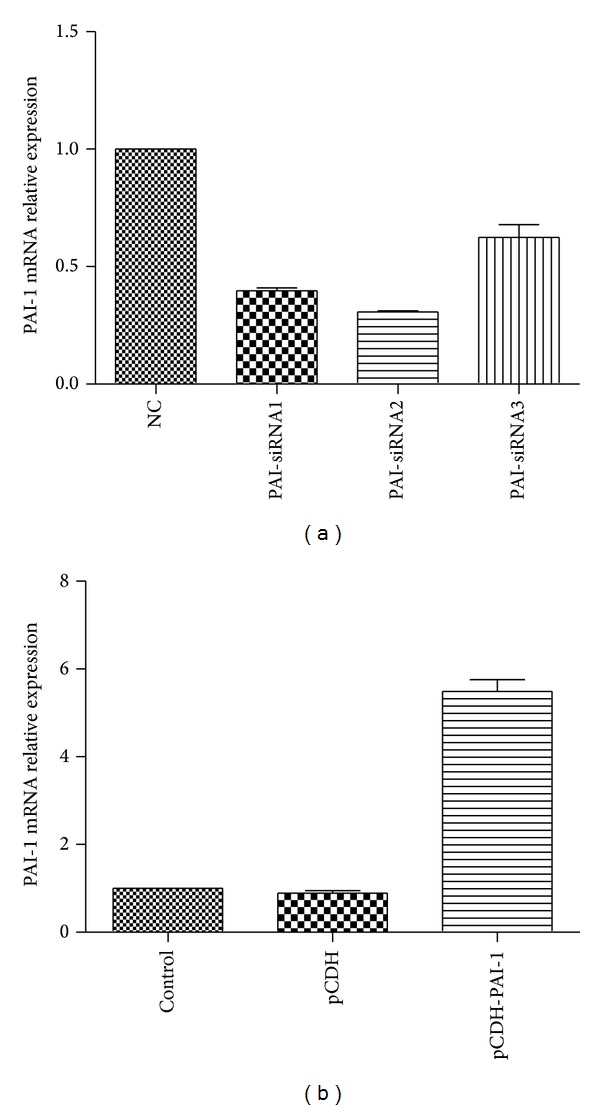
The PAI-1 gene was successfully regulated by transfection with siRNA and pCDH-PAI-1. (a) siRNA knockdown; (b) overexpression by pCDH-PAI-1.

RT-PCR was used to verify the effect of transfection on PAI-1 gene expression in NR8383 cells. As shown in Figure 1(a), transfection with PAI-1 siRNA sequences resulted in reduced expression of the PAI-1 gene in NR8383 cells. From the three sequences studied, PAI-1siRNA2 was the most effective with 69.29% downregulation of PAI-1 mRNA levels observed. Therefore, this sequence was chosen for further experiments. Likewise, transfection with the plasmid pCDH-PAI-1 resulted in 6-time increase in PAI-1 mRNA levels compared with untransfected NR8383 cells or cells transfected with the control plasmid pCDH (Figure 1(b)).

### 3.2. PAI-1 Levels Affect the Release of Proinflammatory Cytokines in LPS-Induced NR8383 Cells

**Figure 2:**
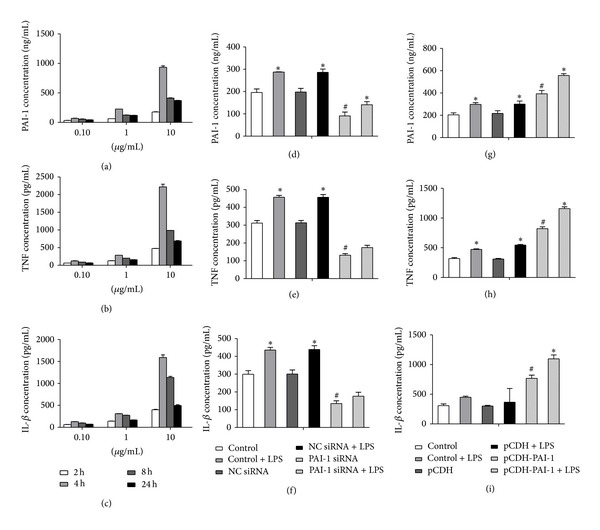
PAI-1, TNF-*α*, and IL-1*β* protein expression in LPS-induced NR8383 cells expressing different levels of PAI-1. Levels of PAI-1 (a, d, g), TNF alpha (b, e, h), and IL-1b (c, f, i) were assessed by ELISA. (a)–(c) Normal NR8383 cells induced with LPS for 2, 4, 8, and 24 h; (d)–(f) control NR8383 cells and cells transfected with PAI-1 siRNA (4 h LPS induction); (g)–(i) control NR8383 cells and cells transfected with the pCDH-PAI-1 plasmid (4 h LPS induction).

In a preliminary experiment, three concentrations of LPS (0.1, 1, and 10 *μ*g/mL) were tested to induce inflammation in NR8383 cells at different incubation times (2, 4, 8, and 24 h). As shown in Figures 2(a)–2(c), TNF-*α*, IL-1*β*, and PAI-1 levels were highest in supernatants collected from cells treated for 4 h with 10 *μ*g/mL LPS. Therefore, 4 h and 10 *μ*g/mL LPS were chosen for subsequent experiments.

Next we evaluated the TNF-*α*, IL-1*β*, and PAI-1 protein levels in supernatants collected from normal NR8383 cells and cells transfected with PAI-1 siRNA induced with 10 *μ*g/mL LPS for 4 h. As expected, the levels of all three proteins increased in control cells after LPS stimulation (Figures 2(d)–2(f), *P* < 0.05). Similar results were obtained when the cells were transfected with the control Si-RNA (NC SiRNA). In contrast, PAI-1 knockdown resulted in lower TNF-*α*, IL-1*β*, and PAI-1 contents in NR8383 cells, compared with control cells (Figures 2(d)–2(f), *P* < 0.05).

An opposite effect was obtained in cells overexpressing PAI-1. As shown in Figures 2(g)–2(i), the expression of PAI-1, TNF-*α*, and IL-1*β* in supernatants was higher than in control cells after transfection with the pCDH-PAI-1 plasmid (*P* < 0.05). After 4 h treatment with LPS, PAI-1 and TNF-*α* amount in supernatants were significantly higher compared to noninduced cells. Although showing the same trend, IL-1*β* levels were not statistically different (*P* > 0.05).

### 3.3. PAI-1 Levels Affect mRNA and Protein Levels of TLR4 and MyD88 in LPS-Induced NR8383 Cells

**Figure 3:**
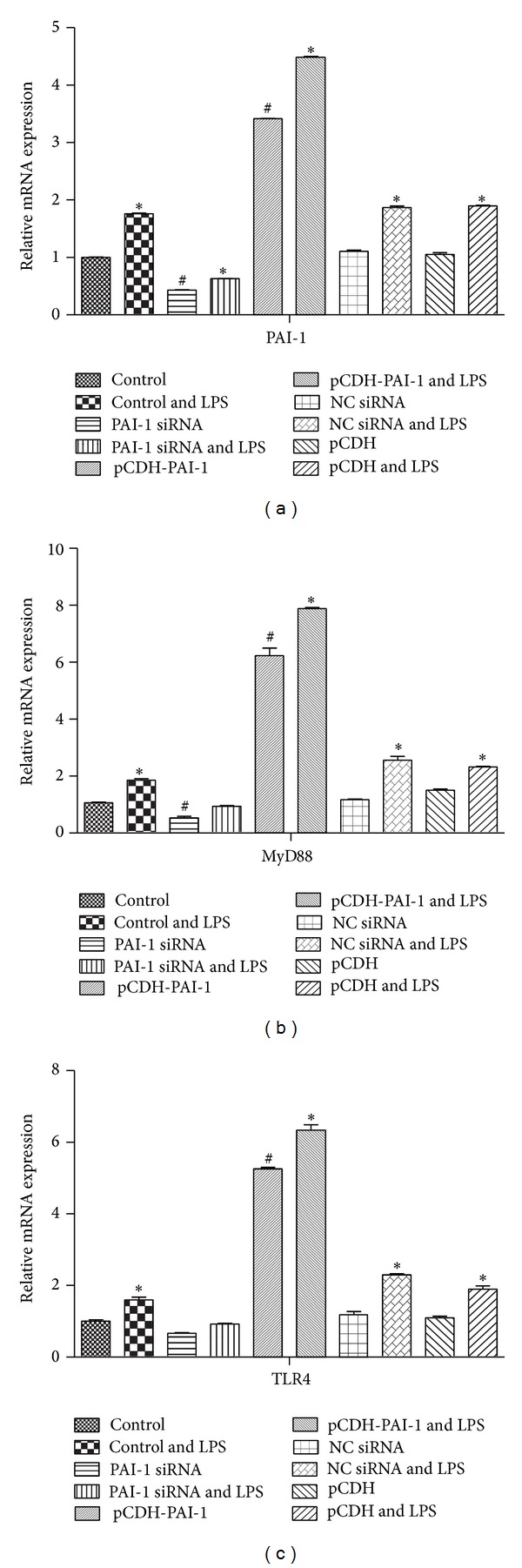
TLR4, MyD88, and PAI-1 mRNA levels in LPS-induced NR8383 cells expressing different levels of PAI-1. (a) Levels of TLR4, (b) MyD88, and (c) PAI-1 were derived with GAPDH as internal control.

LPS treatment resulted in increased gene expression of TLR4, MyD88, and PAI-1 (*P* < 0.05) as shown in Figures 3(a)–3(c). Upon PAI-1 knockdown in NR8383 cells, statistically significant lower TLR4 and MyD88 mRNA levels were obtained compared with control cells (*P* < 0.05). Interestingly, overexpression of PAI-1 resulted in increased gene expression of TLR4 (*P* < 0.05). Similar results were obtained for MyD88 and PAI-1 (Figure 3).

**Figure 4:**
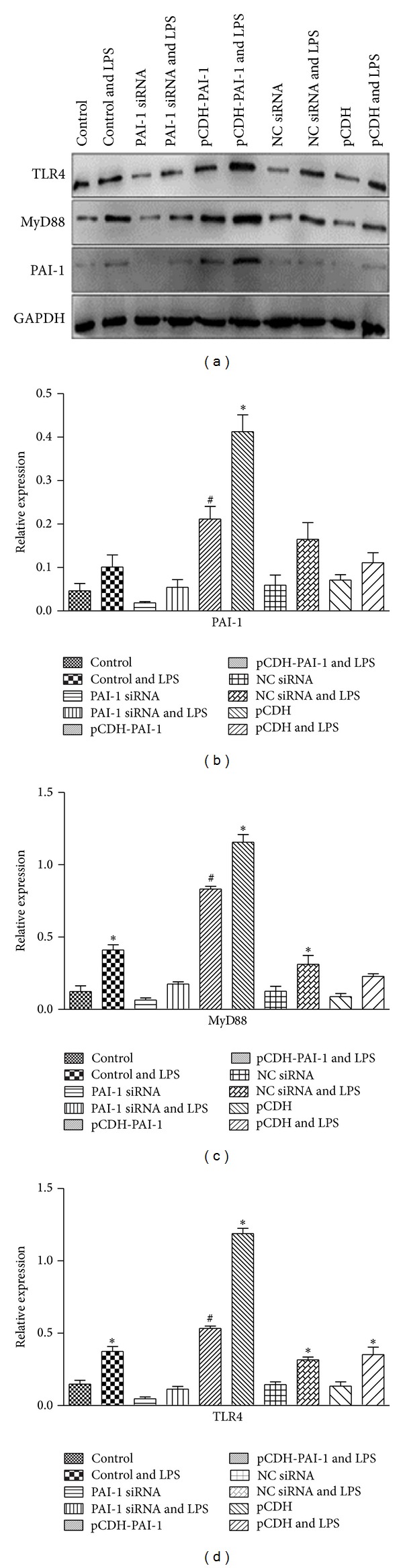
TLR4, MyD88, and PAI-1 protein levels in LPS-induced NR8383 cells expressing different levels of PAI-1. (a) Western blot autoradiography data, (b) levels of TLR4, (c) MyD88, and (d) PAI-1 were derived with GAPDH as internal control.

Similar results were obtained at the protein level.  Figure 4(a) shows representative western blot autoradiograms of samples collected from normal NR8383 cells and cells transfected with PAI-1 siRNA or pCDH-PAI-1 and induced with 10 *μ*g/mL LPS for 4 h. Treatment with LPS resulted in increased expression of TLR4 (*P* < 0.05) as shown in Figures 4(a) and 4(d). Upon PAI-1 knockdown in NR8383 cells, lower TLR4 levels were obtained although without statistical significance compared with control cells. Interestingly, overexpression of PAI-1 led to higher amounts of TLR4 (*P* < 0.05). Similar results were obtained for MyD88 and PAI-1. However, for these two proteins, statistically significant differences were obtained, with PAI-1 siRNA decreasing the expression of MyD88 and PAI-1 and pCDH-PAI-1 enhancing the levels of these proteins (Figures 4(a)–4(c)).

### 3.4. Effect of PAI-1 Levels on mRNA and Protein Expression of Autophagy Markers in LPS-Induced NR8383 Cells

**Figure 5:**
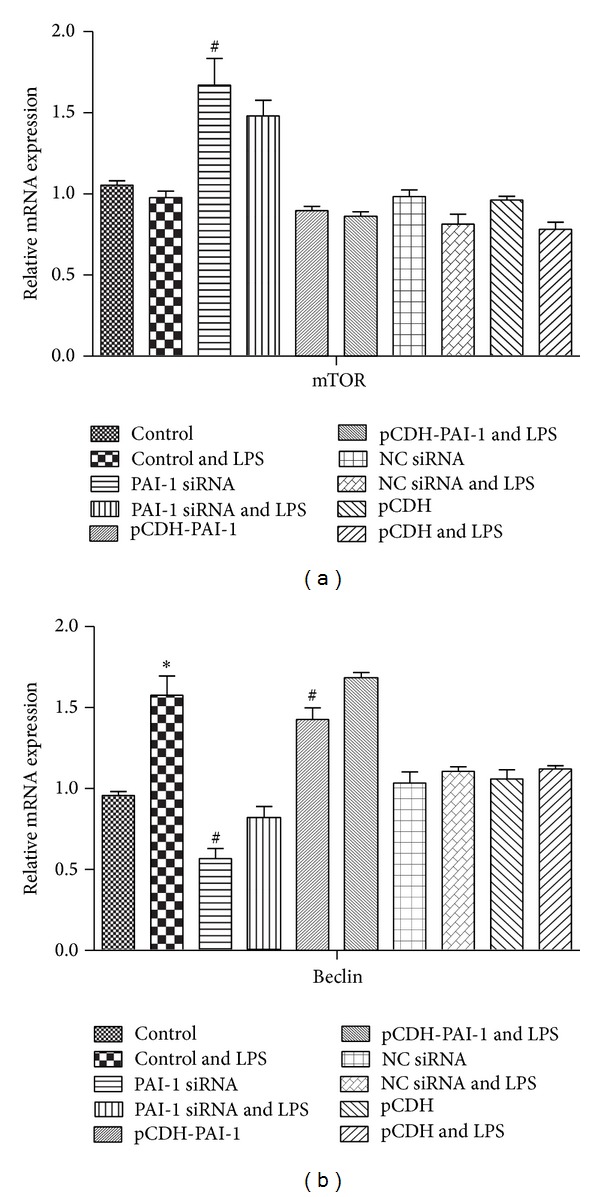
Beclin and mTOR mRNA levels in LPS-induced NR8383 cells expressing different levels of PAI-1. (a) Levels of mTOR and (b) Beclin were derived with GAPDH as internal control.

**Figure 6:**
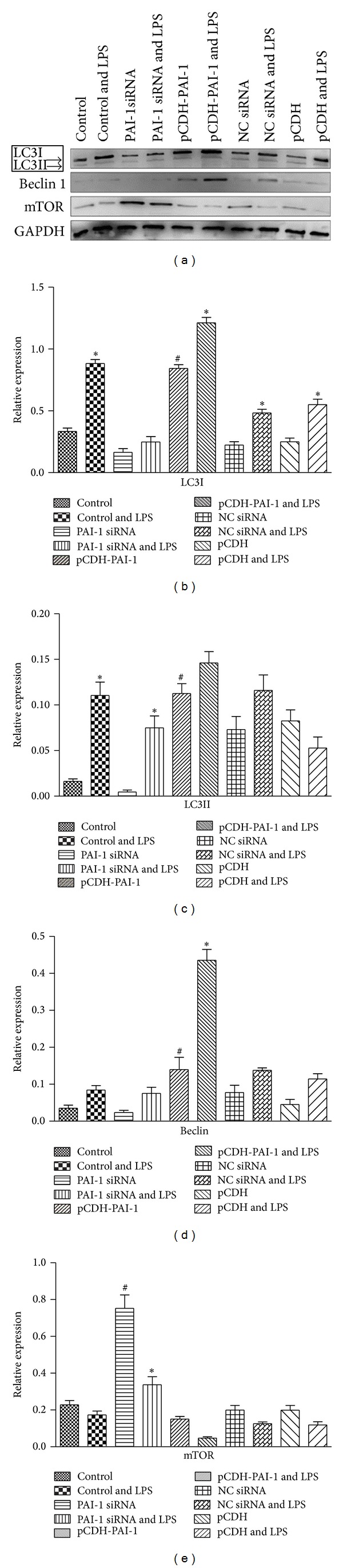
Effect of PAI-1 levels on protein levels of the autophagy markers LC3 and Beclin as well as the autophagy inhibitor mTOR. (a) Western blot autoradiography data, (b) levels of LC3I, (c) LC3II, (d) mTOR, and (e) Beclin were derived with GAPDH as internal control.

When mRNA and protein levels of mTOR (an autophagy inhibitor) were assessed in unstimulated cells, PAI-1 siRNA transfection resulted in increased expression (*P* < 0.05) but no significant change was obtained after transfection with pCDH-PAI-1 (Figures 5(a), 6(a), and 6(d)). After LPS treatment, the expression of mTOR decreased compared to unstimulated cells, but a statistically significant difference was obtained only at the protein level with PAI-1 siRNA transfection (*P* < 0.05).

In NR8383 cells treated with LPS, levels of PAI-1 affected the protein expression of the autophagy markers LC3I and II. As shown in Figures 6(a)–6(c), the levels of LC3I and II were decreased and increased, respectively, after transfection with PAI-1 siRNA and pCDH-PAI-1.

For another autophagy marker, Beclin1, RT-PCR analysis showed that its expression decreased with a statistically significant difference at *P* < 0.05 (Figure 5(b)) after PAI-1 silencing. However, PAI-1 siRNA did not affect protein expression (Figures 6(a) and 6(e)). On the other hand, RT-PCR and western blot data showed that transfection with pCDH-PAI-1 significantly increased the expression of Beclin1 compared with normal control cells at *P* < 0.05 (Figures 5(b), 6(a), and 6(e)). Upon LPS induction, Beclin1 mRNA and protein levels were elevated in comparison with unstimulated pCDH-PAI-1 transfected cells (*P* < 0.05) as shown by RT-PCR (Figure 5(b)) and western blot (Figures 6(a) and 6(e)).

### 3.5. GFP-LC3 Transfection Confirms the Effect of PAI-1 Levels on LC3 Expression by NR8383 Cells

**Figure 7:**
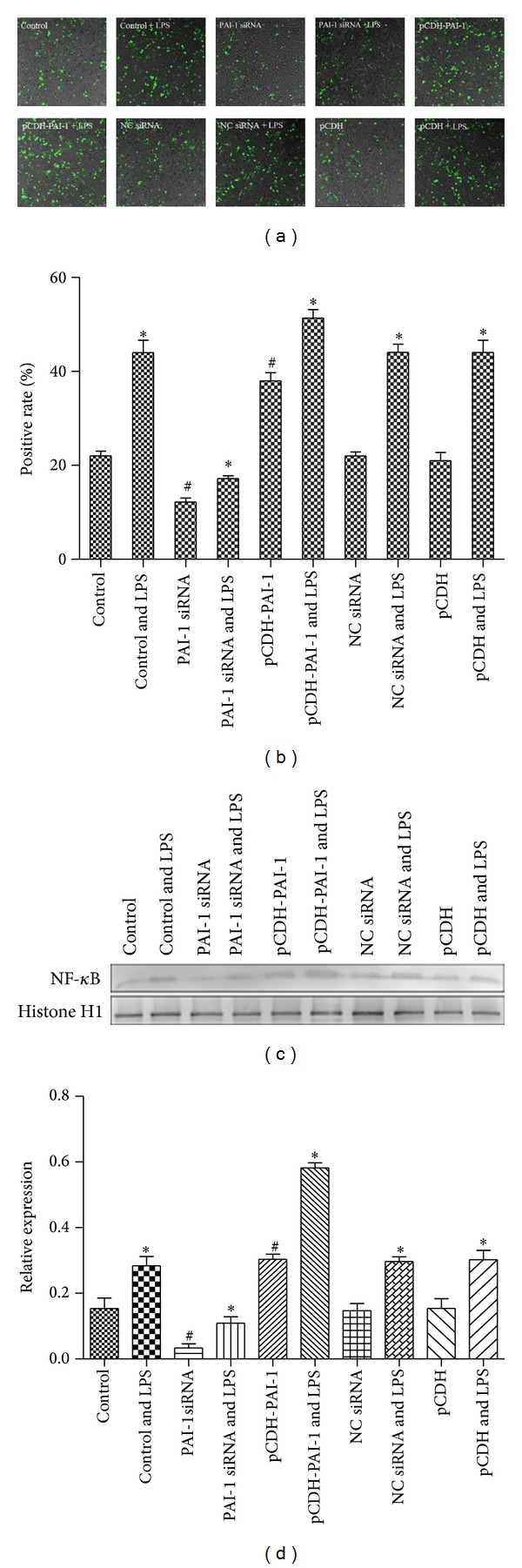
Effect of PAI-1 levels on rate of LC3-expressing cells and NF-*κ*B. (a) Representative images of LC3 positive cells in different groups; (b) overexpression and knockdown of PAI-1 affect the rate of LC3 positive cells; (c) western blot autoradiography data with 1 (control), 2 (control and LPS), 3 (PAIsiRNA), 4 (PAIsiRNA and LPS), 5 (pCDH-PAI-1), 6 (pCDH-PAI-1 and LPS), 7 (NC siRNA), 8 (NC siRNA and LPS), 9 (pCDH), and 10 (pCDH and LPS); (d) relative NF-*κ*B protein levels derived with Histone H1 as internal control.

NR8383 cells were transfected with the GFP-LC3 plasmid and positive cells expressed the green florescent protein which indicated autophagosome formation. We found that LPS induction resulted in an increase in GFP-LC3 positive cells (*P* < 0.05) as shown in Figures 7(a) and 7(b). Interestingly, transfection with pCDH-PAI-1 enhanced the rate of GFP-LC3 positive cells, whereas PAI-1 knockdown decreased this rate, compared with control cells (*P* < 0.05).

### 3.6. Effect of PAI-1 Levels on NF-*κ*B Protein Levels in LPS-Induced NR8383 Cells

As shown in Figures 7(c) and 7(d), NF-*κ*B protein levels were significantly increased after transfection with pCDH-PAI-1 in NR8383 cells, in comparison with control cells (*P* < 0.05). Conversely, PAI-1 knockdown reduced the levels of NF-*κ*B (Figures 7(c) and 7(d)). LPS induction resulted in increased expression of NF-*κ*B in all groups, compared with unstimulated cells (*P* < 0.05) as shown in Figures 7(c) and 7(d).

## 4. Discussion

PAI-1 is a key regulator for fibrinolysis and coagulation, counteracting plasminogen activators (PAs), such as urokinase (uPA) and tissue-type PA (tPA) [20]. In addition, PAI-1 acts as an acute phase protein (APP) during APR such as acute lung injury (ALI), inflammation, and sepsis [7, 21–24] and is closely associated with poor outcome in ALI patients [25, 26]. Since autophagy is also a known mechanism that regulates cell death and inflammation, we assessed the relationship between PAI-1 and autophagy in the regulation of inflammation. We chose rat alveolar macrophages, important inflammatory effector cells in this work, and used LPS to induce inflammatory reactions. As shown above, LPS stimulated NR8383 cells expressed and released higher amounts of TNF-*α*, IL-1*β*, and PAI-1 compared with control cells. In addition, we demonstrated that overexpression and knockdown of the PAI-1 gene increased and decreased the inflammation markers, respectively.

The TLR4 signal transduction pathway plays a critical role in LPS intracellular signal transduction and MyD88 is a key component in TLR/NF-*κ*B pathway of LPS-induced inflammatory reactions [27]. Interestingly, in this study, downregulation of PAI-1 levels using PAI-1 siRNA reduced the expression of TLR4, MyD88, and NF-*κ*B, while PAI-1 overexpression showed opposite results. These findings indicated that during LPS-induced inflammatory reaction, PAI-1 promotes inflammation through the TLR4/MyD88/NF-*κ*B pathway. This may be particularly important in ARDS. Indeed, PAI-1 deficiency was recently shown to exacerbate LPS-induced ALI by enhancing toll-like receptor 4 signaling pathway [28].

Unstimulated alveolar macrophages are profibrinolytic. Primary isolates of human alveolar macrophages display PA activity and degrade a fibrin matrix in the presence of plasminogen [8]. By contrast, stimulated alveolar macrophages are antifibrinolytic; human alveolar macrophages exposed to endotoxin inhibit fibrinolysis through an increased PAI-1 activity [10]. Increased PAI-1 activity has also been observed in macrophages isolated from patients with lung disease [29], indicating a decrease in the fibrinolytic activity of alveolar macrophages through decreased PA activity and increased PAI-1 activity when the lung is exposed to injurious stimuli.

Next we investigated whether these effects on inflammation were related to autophagy, a cell process that plays multiple roles in organism survival and homoeostasis, for example, by self-digestion induced by nutrient deprivation or increased metabolic demand [30, 31]. The microtubule-associated protein LC3 (or Atg8) is a unique molecular marker for autophagosomes. The conversion of LC3-I (cytosolic form) to LC3-II (membrane-bound lipidated form) is widely recognized as an early trait of autophagy activation [32]. The appearance of punctate staining in green fluorescence protein (GFP-) LC3-expressing cells and tissues is widely used as a “gold standard” index of autophagosome formation [33]. As shown above, LC3-I and LC3-II were all increased upon LPS induction. PAI-1 siRNA transfection resulted in decreased expression of both LC3-I and LC3-II, whereas PAI-1 upregulation increased the amounts of both LC3 variants.

Beclin1 is an analog of the yeast autophagy gene ATG6, which is composed by yeast P13K complex; the interaction between Beclin1 and P13K induces autophagy under conditions of starvation in mammal cells. Therefore, enhanced expression of Beclin1 constitutes a measure of autophagy increase [34]. In addition, Beclin1 participates in the formation of autophagosomes [35]. Recent studies have shown that one mode of action of proinflammatory cytokines consists of inducing the autophagy pathways, an effect counteracted by anti-inflammatory cytokines [36]. Although cellular metabolic pathways have been extensively studied over decades, autophagy is relatively less understood. Its primary function in normal cells is to complement the ubiquitin-proteasome systems by assisting in maintaining the fine balance between protein synthesis, organelle biogenesis, and degradation [37]. In addition, autophagy also contributes to the removal of damaged and potentially toxic proteins that are prone to form aggregates. We found that the expression of Beclin1 was increased in NR 8383 cells induced by LPS. Knockdown and overexpression of PAI-1 resulted in decrease and increase of Beclin1, respectively. These findings indicate that PAI-1 regulates autophagy in NR 8383 cells.

mTOR is a key negative regulator of autophagy although the mechanism underlying this effect is not well understood [38]. Recent evidence, however, suggests that mTOR may not be the sole regulator of autophagy, suggesting the possible existence of other pathways that bypass the regulatory effect of this protein. For instance, an increase in cellular ammonia levels activates the autophagy machinery, but in an mTOR independent manner [39]. Our data showed a decrease in mTOR in LPS-induced NR8383 cells. Upon PAI-1 knockdown, mTOR expression was increased, while upregulation of PAI-1 resulted in decreased expression of mTOR. These findings confirm the proautophagy effects of PAI-1.

It is accepted that autophagy influences the immune system during pathogen clearance by regulating antigen presentation, lymphocyte development, and proinflammatory cytokine production [17]. However, the mechanism by which autophagy regulates cytokine secretion remains poorly understood. Our data indicated that upregulation of PAI-1 increases the expression of inflammatory cytokines in macrophage through enhancement of autophagy, with PAI-1 knockdown showing the opposite effect. Overall, our data suggest that PAI-1 regulates inflammation in macrophages likely through autophagy modulation. These findings provide a basis for the use of PAI-1 as a molecular target for the treatment of inflammatory diseases.

## Figures and Tables

**Table 1:** The sequences of small interfering RNA used for plasminogen activator inhibitor-1 (PAI-1) silencing.

PAI1-siRNA1	Sense	5′-CCAGAUUCAUCAUCAAUGATT-3′
	Antisense	5′-TTGGUCUAAGUAGUAGUUACU-3′

PAI1-siRNA2	Sense	5′-GGCACACCAAAGGUAUGAUTT-3′
	Antisense	5′-TTCCGUGUGGUUUCCAUACUA-3′

PAI1-siRNA4	Sense	5′-GGCACACCAAAGGUAUGAUTT-3′
	Antisense	5′-TTCCGUGUGGUUUCCAUACUA-3′

NC-siRNA	Sense	5′-UUCUCCGAACGUGUCACGUTT-3′
	Antisense	5′-TTAAGAGGCUUGCACAGUGCA-3′

**Table 2:** Primer sequences used in real-time polymerase chain reaction.

PAI-1	Forward	5′-TCTGGGAAAGGGTTCACTTTACC-3′
	Reverse	5′-GACACGCCATAGGGAGAGAAG-3′

TLR4	Forward	5′-GCCTTTCAGGGAATTAAGCTCC-3′
	Reverse	5′-GATCAACCGATGGACGTGTAAA-3′

MyD88	Forward	5′-TCATGTTCTCCATACCCTTGGT-3′
	Reverse	5′-AAACTGCGAGTGGGGTCAG-3′

LC3	Forward	5′-GACTTCCGGAAAGCTCTGCT-3′
	Reverse	5′-ACCAGCATCGTAGAGGGTCT-3′

Beclin1	Forward	5′-GCCTCTGAAACTGGACACGA-3′
	Reverse	5′-CTTCCTCCTGGCTCTCTCCT-3′

mTOR	Forward	5′-TCCTGAAGAACATGTGCGAG-3′
	Reverse	5′-CCAAAGTACAAGCCAGAGGC-3′

GAPDH	Forward	5′-AATGCATCCTGCACCACCAA-3′
	Reverse	5′-GATGGCATGGACTGTGGTCA-3′

## Abbreviations

(PAI-1): Plasminogen activator inhibitor-1
(PA): Plasminogen activator
(tPA): Tissue-type plasminogen activator
(uPA): Urokinase-type plasminogen activator
(ARDS): Acute respiratory distress syndrome
(ALI): Acute lung injury
(LPS): Lipopolysaccharide
(ELISA): Enzyme-linked immunosorbent assay
(TNF-*α*): Tumor necrosis factor-*α*
(IL-1*β*): Interleukin-1*β*
(TLR4): Toll-like receptor 4
(MyD88): Myeloid differentiation primary response 88
(LC3): Light Chain 3
(mTOR): Mammalian target of rapamycin
(IFN-*γ*): Interferon-*γ*
(NF-*κ*B): Nuclear factor-*κ*B
(RT-PCR): Reverse transcriptase-polymerase chain reaction.
